# The heterogeneous effects of perceptions of digital and intelligent technologies on multidimensional political identity among college students: a study of multiple parallel mediations from a stratified cluster perspective

**DOI:** 10.3389/fpsyg.2026.1794810

**Published:** 2026-10-22

**Authors:** Qian Hou

**Affiliations:** School of Marxism, Shandong University of Technology, Zibo, China

**Keywords:** dimensional hierarchy mechanism, heterogeneity effects, multiple parallel mediation model, perceptions of digital and intelligent technologies, political identity, political socialization of youth

## Abstract

**Introduction:**

The digital transformation of young people’s political socialization is a complex process fraught with tensions. Existing Western theories such as the theory of technological neutrality and the theory of algorithmic polarization—struggle to fully account for state-led digital ecosystems, while domestic Chinese research often treats political identity as a homogeneous concept. Based on this, this study aims to uncover the stratified and heterogeneous mechanisms through which perceptions of digital and intelligent technologies shape the multidimensional political identities of Chinese college students.

**Methods:**

Drawing on survey data from 4,094 Chinese college students, this study expands the classic six-dimensional political identity framework into an eight-dimensional system (adding the emotional and institutional dimensions) and categorizes these dimensions into three clusters: the Emotional–Traditional Cluster, the Institutional–Performance Cluster, and the Party Identity Cluster. A bootstrap multiple parallel mediation model with bias correction using 5,000 repeated samples was employed to test five transmission pathways.

**Results:**

The results reveal significant differences: perceptions of political participation and rights exert significant positive mediating effects, while perceptions of interests generally exhibit an inhibitory (masking) effect. The Institutional–Performance Cluster primarily exerts its influence through the political participation pathway (mean pathway effect *β* = 0.107); its total mediation effect (*β* = 0.260) is approximately 2.4 times that of the Emotional Traditional Cluster (*β* = 0.108); political communication exerts an appreciable effect only on the Institutional–Performance Cluster.

**Discussion:**

The findings of this study qualify the claims of universality made by Western theories such as algorithmic polarization and technological neutrality. By constructing a causal chain of “digital-intellectual cognition—multiple mediation— stratified identity,” this study provides a non-Western empirical model for understanding the political socialization of youth in the algorithmic era.

## 1 Introduction

The impact of the digital revolution on the political socialization of youth is not a simple, linear process of either strengthening or weakening, but rather a complex process fraught with tensions. In the context of Western multiparty systems, research often focuses on the “echo chamber” effect caused by algorithmic polarization and the crisis of civic trust (da Silva Neto and Chiarini, 2021). However, within China’s state-led digital ecosystem, digital and intelligent technologies have reshaped the structure of young people’s political identities through unique technopolitical mediation mechanisms. This reshaping is not only reflected in the digital migration of participation forms but is more profoundly manifested in the interactive restructuring of cognitive pathways, emotional mobilization, and institutional trust.

### 1.1 The Western perspective: the theoretical tension between technological neutrality and algorithmic polarization

Western academic discourse on the impact of digital technology on political attitudes is characterized by a dichotomy. The theory of technological neutrality holds that technology itself has no inherent moral bias, and its social consequences depend entirely on how humans use it (Miller, 2020). In contrast, the theory of algorithmic polarization argues that algorithmic curation traps users in “filter bubbles” and “echo chambers,” fundamentally eroding political cohesion (Wolfowicz et al., 2021). Empirical research confirms that algorithms limit users’ exposure to diverse viewpoints and exacerbate social polarization (Kelm et al., 2023). Furthermore, opaque algorithmic governance may trigger a “legitimacy deficit” (König and Wenzelburger, 2021), and some scholars even warn that the digital age is falling into a vicious cycle where “division breeds division” (Ford, 2025).

However, the aforementioned theories are all rooted in the context of Western multiparty competition. Recent research has begun to reflect on the limitations of Western-centric perspectives, pointing out that non-Western, state-led digital ecosystems may exhibit entirely different operational logics (Trenz, 2023). In China, the state actively deploys AI-driven platforms (such as smart governance apps) to disseminate policy narratives and foster social consensus (Zeng, 2020; Zhu and Wang, 2024), providing a crucial comparative case for testing Western hypotheses on polarization.

### 1.2 Progress in research on political identity among Chinese youth and the limitations of “dimensional homogeneity”

Although research on the political socialization of Chinese youth has evolved, significant blind spots remain.

First, while the measurement framework has become multidimensional, its operationalization tends toward homogeneity. Shi et al. (2014) established a classic framework encompassing dimensions such as identity, institutions, political parties, and policies. However, subsequent empirical studies often combine these dimensions into a single “total political identity score” for inclusion in regression analyses (Zhang and Guo, 2020; Tao and Zhang, 2022), implicitly assuming—without testing—that “the effects of the variables are equal across all dimensions.”

Second, research on behavioral mechanisms focuses on participation, but the effects are complex. Most studies confirm that online political participation helps enhance identity (Zheng, 2017; Zhou and Liu, 2018). However, the effects of digital media are not uniformly positive; digital fragmentation and “smartphone dependency” may lead to behavioral deviance (Wang and Luo, 2018), and these effects vary depending on the type of media and content orientation (Liu et al., 2023; Yang, 2020).

Finally, exploration of mediating mechanisms remains limited. Although some studies have pointed out that political trust influences identity through perceived performance (Tao and Zhang, 2022), most models still employ single or chained mediation, with very few examining the complete chain of “digital technology → multiple parallel mediators → clusters of specific identity dimensions.”

In summary, the existing literature treats political identity as a homogeneous construct, failing to distinguish between the differentiated responses to technological stimuli exhibited by affective-traditional identities (such as a sense of cultural belonging) and institutional-performance-based identities (such as policy satisfaction). This assumption of “dimensional homogeneity” obscures the complex mechanisms through which digital technology shapes identity.

### 1.3 Theoretical framework and research approach

Based on the previous discussion of theoretical tensions in Western scholarship and limitations in Chinese research, this study constructs an exploratory analytical framework of “perceptions of digital and intelligent technologies—multiple mediators—stratified, multidimensional political identity” (see Figure 1). We do not presuppose a unidirectional linear relationship but rather argue that the shaping of political identity by digital technologies is a process involving multiple pathways of influence.

**Figure 1:**
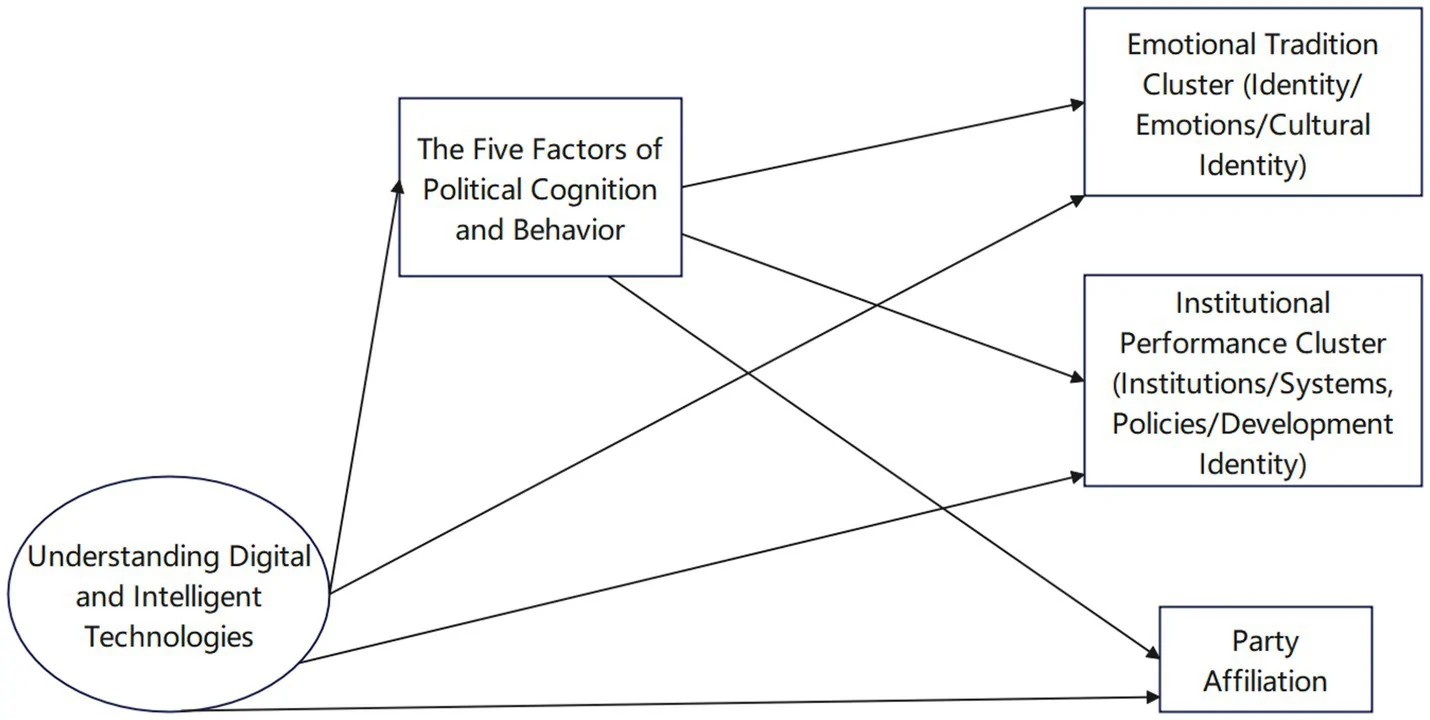
An exploratory framework for the multiple parallel mediating effects of perceptions of digital and intelligent technologies on political identity. “The Five Factors of Political Cognition and Behavior” specifically refers to perceptions of rights, perceptions of interests, political participation, political communication, and political satisfaction.

Addressing the long-standing limitation of “dimensional homogeneity” in domestic research (Shi et al., 2014), this study focuses on introducing a dimensional stratification perspective. We classify the eight dimensions of identity into two major psychological clusters: the “emotional-endogenous” cluster (identity, emotion, and cultural identity) and the “performance-exogenous” cluster (institutional, systemic, policy, and developmental identity). Here, “endogenous” specifically refers to the fact that the core driving force of this type of identity stems from individuals’ innate emotional connections and the inheritance of cultural genes, and is characterized by stability and non-utilitarianism. Social identity serves as a bridge connecting group membership with policy-related emotions (Hornung and Bandelow, 2025), and the formation of identities within the “emotional-endogenous” cluster relies more heavily on a deep bond between emotions and identity. “Exogenous” refers to the fact that the core driving force of this type of identity stems from feedback on governance performance in the external environment and rational cost–benefit assessments, characterized by dynamism and instrumentalism. Individuals’ responses to policies or institutions do indeed involve a cognitive evaluation process, in which they assess whether policies align with their values, interests, and group norms (Schaefer et al., 2024); the “performance-exogenous” cluster embodies the characteristics of such rational construction. Political party identity, in turn, reflects political stances and perceptions. This classification aims to clarify the heterogeneous logic underlying different types of political identity in response to digital stimuli. The core objective of this study is to investigate the impact of perceptions of digital and intelligent technologies on college students’ multidimensional political identities. In other words, are there significant differences in the transmission mechanisms through which perceptions of digital and intelligent technologies influence the identities categorized above?

Based on this framework, this study will employ a multiple parallel mediation model to investigate and systematically deconstruct how perceptions of digital and intelligent technologies influence political identity through five mediating factors. Figure 1 illustrates the influence pathways tested in this study.

## 2 Data and methods

### 2.1 Sample and data collection

The subjects of this study are full-time undergraduate students in China. As a “digital native” group, college students are long-term, frequent users of various digital and intelligent platforms. At the same time, they are in a critical stage of youth when political identity is stabilizing and taking shape, making them an appropriate sample for exploring the mechanisms through which digital and intelligent technologies influence the political socialization of young people. The survey period spanned February through August 2025. A nationwide random sample was employed, covering dimensions such as gender, place of origin, and institution type (“Double First-Class” universities, local comprehensive undergraduate institutions, and higher vocational colleges).

The questionnaire was administered entirely online in a standardized, anonymous format. The duration of a single session was kept between 20 and 45 min, with two research assistants—both having undergone standardized training—available online to answer questions simultaneously. After questionnaire collection, standardized rules for excluding invalid samples were applied: questionnaires with response times under 10 min, those where the same option was selected for five or more consecutive questions, and those with extensive missing data for core latent variables were excluded. Of the 4,182 questionnaires initially collected, 4,094 were deemed valid after screening, resulting in a valid response rate of 97.9%.

This random sampling was used solely to enhance the representativeness of the sample. In all subsequent regression and multiple parallel mediation models, only two demographic characteristics—gender and place of origin—were included as control variables; institution, discipline, and grade level were not included in the statistical models. See Table 1 in the main text for the detailed demographic distribution of the sample.

**Table 1:** Demographic characteristics of the sample (*N* = 4,094).

Variable	Category	Frequency	Valid percentage (%)
Gender	0 (Female)	2,267	55.4
	1 (Male)	1,827	44.6
Hometown Origin	0 (Urban)	2,977	72.7
	1 (Rural)	1,117	27.3

#### 2.1.1 Ethical statement

This study was exempted from formal ethical review by the Research Ethics Committee of Shandong University of Technology. All research participants were undergraduate students aged 18 or older; no minors were included in the sample, and the research posed an extremely low risk. All participants read the full informed consent form online before completing the survey; their consent took effect upon checking the online consent box, and they were free to voluntarily withdraw from the study at any time. Data collection and analysis were conducted anonymously and de-identified throughout the process, and the data was stored in encrypted form.

### 2.2 Measurement

The measurement system in this study is divided into four major modules: demographic control variables, independent variables, mediating variables, and dependent variables. All scales uniformly employ a 5-point Likert scale, and reverse-scored items are uniformly converted using a 6–raw score scale to ensure consistency in scoring direction.

The study employs a mixed-methods measurement paradigm combining multi-item scales and single-item measures: multi-item scales were designed for eight constructs—identity, emotional identification, institutional identification, systemic identification, digital intelligence and technology awareness, rights awareness, political participation, and political communication (totaling 28 items); Six constructs—political party identification, cultural identification, policy identification, development identification, perception of interests, and political satisfaction—were measured using single-item scales following item purification through preliminary testing. For details on the complete scale items, adaptation sources, and records of preliminary testing and purification, please refer to the Supplementary material (Supplementary Tables S1 and S2) of this paper.

#### 2.2.1 Demographic control variables

The model includes only two demographic characteristics as control variables: gender and place of origin.

#### 2.2.2 Dependent variable: eight-dimensional political identity

Building upon the six-dimensional maturity framework of Chinese citizens’ political identity proposed by Shi et al. (2014), and incorporating the characteristics of contemporary college students’ digital political behavior, two new dimensions—emotional identity and institutional identity—have been added. These correspond to the emotional-symbolic and institutional-performance levels of political identity, respectively. The definitions and measurement formats for each dimension are as follows: Identity (3 items): Measures college students’ sense of belonging to the Chinese nation and the national community, such as “As a Chinese person in the new era, I feel very proud”; Emotional Identity (3 items): Measures an individual’s emotional connection to the motherland, the nation, and political parties, such as “How do you feel about your motherland?”; Institutional Identity (3 items): Measures understanding of and value recognition toward China’s political system, such as “To what extent do the values embodied by the political system of socialism with Chinese characteristics align with your own?”; Systemic Identity (3 items): Measures recognition of the achievements of the socialist system with Chinese characteristics, such as “Do you believe that ‘since the reform and opening-up, China’s political system has greatly promoted the country’s development’?”; Party, Cultural, Policy, and Development Identity (1 item each): Single-indicator anchored questions that measure, respectively, approval of the Party’s leadership, cultural pride, support for national policies, and confidence in development through reform and opening-up.

#### 2.2.3 Independent variable: perceptions of digital technologies

This study employed a self-developed 7-item scale to comprehensively measure college students’ overall understanding of digital intelligence technologies. Drawing on the DigComp 2.1 digital competence framework (Mieg et al., 2023) and the core dimensions identified in related studies (Gutiérrez-Ángel et al., 2022), and taking into account the practical concerns regarding digital literacy among Chinese college students (Liang et al., 2025), the scale covers seven dimensions: technology adoption, development trends, security and ethics, impact on daily life, social impact, algorithmic risks, and literacy enhancement. A representative item is “How well do you understand digital intelligence technologies?” To operationalize this construct as an integrated competency model, this study adopted an equal-weighting approach, incorporating the average scores of the seven items into the model. This method preserves the theoretical integrity of the unified construct while avoiding arbitrary statistical weights imposed by sample-specific variances. The scale demonstrated good reliability (Cronbach’s *α* = 0.889) and excellent item-total correlation.

#### 2.2.4 Mediating variables

Five parallel mediators were established to construct the “Digital Technology Cognition — Perception / Participation — Political Identity” transmission pathway: Political Participation, Political Communication, and Perception of Rights (3 items each)—multi-item scales measuring digital political participation behaviors, perceptions of political communication, and perceptions of political rights, respectively; Perception of Interests and Political Satisfaction (1 item each)—single-indicator measures, where Perception of Interests is a reverse-scored anchor item that requires score conversion before inclusion in the analysis.

### 2.3 Psychometric properties

#### 2.3.1 Internal consistency reliability

The results for the reliability of all multidimensional scales show that the overall Cronbach’s α for the entire scale is 0.925; for Emotional Identity and Cognitive Perception of Digital and Intelligent Technologies, *α* > 0.8, indicating excellent reliability; for Identity, Rights, Political Participation, and Communication, α ranges from 0.71 to 0.77, indicating good reliability; and for Institutional and Systemic Identity, α ranges from 0.65 to 0.67, meeting measurement standards. All items had a CITC score greater than 0.3; there were no invalid items, and all were retained.

#### 2.3.2 Structural validity and data fit

KMO and Bartlett’s sphericity tests were conducted on the 28 multi-item scales, yielding KMO = 0.941, chi-square = 56,721.164, df = 378, *p* < 0.001, indicating that the data are fully suitable for factor analysis. Further exploratory factor analysis was conducted, yielding four common factors with eigenvalues greater than 1, explaining a cumulative variance of 56.09%, which meets the 50% passing standard; the variance of the first factor before rotation was 34.62% < 40%, indicating no serious common method bias. The results of confirmatory factor analysis showed that all fit indices for the eight-factor model met the criteria, confirming the validity of the eight-dimensional theoretical structure of political identity. Robust standard errors were used in all regression and mediation models to correct for heteroscedasticity.

#### 2.3.3 Confirmatory factor analysis (CFA) and structural validity

For the eight latent variables—identity, emotional identification, institutional identification, systemic identification, perception of digital intelligence technology, perception of rights, political participation, and political communication—convergent validity tests were conducted by independently constructing single-factor measurement models for each variable. The standardized factor loadings for all items were significant at the *p* < 0.001 level. Key indicators of convergent validity showed that the average variance extracted (AVE) for the eight dimensions was >0.5 and the composite reliability (CR) was >0.7, fully meeting the Fornell-Larcker criteria for convergent validity, indicating good internal consistency of the scale. Among these, the standardized factor loading for a single item in the political communication dimension was 0.580, slightly below the conventional threshold of 0.6. However, this item could not be theoretically omitted, and since the dimension’s overall AVE (0.532) and CR (0.770) both met the criteria, all items were retained in their entirety.

Overall discriminant validity test. The four significant multi-item dimensions of political identity—identity, emotional identification, institutional identification, and systemic identification—were incorporated into the same four-factor reference model. The overall fit results are shown in Table 2: *χ*^2^(48) = 1407.880, CFI = 0.925, TLI = 0.897, SRMR = 0.054, meeting the general fit criteria for social science scales; RMSEA = 0.083, which slightly exceeds the critical threshold of 0.08. Given the characteristics of the very large sample size (4,094 respondents), chi-square-based indices are prone to prematurely rejecting models. Based on a comprehensive assessment of robust fit indices such as CFI and SRMR, the four-factor measurement model is structurally stable and valid, and the four dimensions of political identity demonstrate good discriminant validity, confirming that the multidimensional classification of political identity in this study is supported by reliable measurement.

**Table 2:** Goodness-of-fit indices for the four dimensions of political identity (multi-item).

Fit indices	Value	Acceptable standard	Excellent standard	Evaluation
*χ*^2^(df)	1407.880 (48)	*p* < 0.05	–	Significant
Comparative Fit Index (CFI)	0.925	>0.90	>0.95	Good
Tucker–Lewis Index (TLI)	0.897	>0.90	>0.95	Marginal/Acceptable
Root Mean Square Error of Approximation (RMSEA)	0.083	<0.08	<0.05	Critical
90% CI	[0.079, 0.087]	Upper limit < 0.08	–	Slightly Exceeded
Standardized Root Mean Square Residual (SRMR)	0.054	<0.08	<0.05	Excellent

CFI, Comparative Fit Index; TLI, Tucker–Lewis Index; RMSEA, Root Mean Square Error of Approximation; SRMR, Standardized Root Mean Square Residual; CI, Confidence Interval. Evaluation Criteria: Following conventional standards (e.g., Hu and Bentler, 1999), CFI and TLI values greater than 0.90 indicate acceptable fit, with values greater than 0.95 suggesting good fit. RMSEA and SRMR values less than 0.08 represent acceptable model fit.

### 2.4 Analytical tools and model settings

This study used SPSS 26 and Stata 18 to conduct data analysis. SPSS 26 was used to perform descriptive statistics, reliability tests, exploratory factor analysis, and correlation analysis. Model 4 of the PROCESS package was used to test for multiple concurrent mediating effects; 5,000 bootstrap samples were drawn to calculate indirect effects at the 95% confidence interval, and robust standard errors were employed to address heteroscedasticity. The structural equation modeling module in Stata 18 was used to conduct confirmatory factor analysis to test the validity of the measurement model. All regression models controlled for gender and place of origin.

## 3 Results

A multiple parallel mediation model analysis was conducted based on a valid sample of 4,094 full-time undergraduate students nationwide. All models included gender and place of origin as control variables, and the significance of the mediating effects was tested using the bias-corrected Bootstrap method with 5,000 repetitions. The pairwise correlations among the variables are shown in Table 3.

**Table 3:** Pearson correlation matrix for the variables (*N* = 4,094).

Variable name	dig_m	rig_m	par_m	com_m	ben_m	sat_m	ide_m	emo_m	ins_m	sys_m	par_id	cul_id	pol_id	dev_id
dig_m	–													
rig_m	0.53**	–												
par_m	0.61**	0.59**	–											
com_m	0.62**	0.54**	0.69**	–										
ben_m	−0.32**	−0.38**	−0.26**	−0.26**	–									
sat_m	0.54**	0.47**	0.62**	0.64**	−0.19**	–								
ide_m	0.23**	0.24**	0.24**	0.22**	0.02	0.24**	–							
emo_m	0.23**	0.26**	0.29**	0.25**	0	0.29**	0.51**	–						
ins_m	0.47**	0.42**	0.56**	0.50**	−0.08**	0.46**	0.34**	0.43**	–					
sys_m	0.40**	0.41**	0.50**	0.47**	−0.08**	0.46**	0.40**	0.49**	0.60**	–				
par_id	0.29**	0.31**	0.35**	0.31**	0	0.33**	0.44**	0.51**	0.44**	0.56**	–			
cul_id	0.39**	0.38**	0.43**	0.39**	−0.05**	0.38**	0.38**	0.38**	0.45**	0.51**	0.50**	–		
pol_id	0.49**	0.48**	0.56**	0.54**	−0.11**	0.51**	0.26**	0.30**	0.53**	0.49**	0.36**	0.44**	–	
dev_id	0.38**	0.40**	0.44**	0.43**	−0.03*	0.42**	0.36**	0.36**	0.42**	0.50**	0.48**	0.49**	0.501**	–

Variable key: dig_m = digital intelligence technology cognition; rig_m = rights cognition; par_m = political participation; com_m = political communication; ben_m = benefit cognition; sat_m = political satisfaction; ide_m = identity; emo_m = emotion; ins_m = institution; sys_m = system; par_id = party identification; cul_id = culture; pol_id = policy; dev_id = development). *p < 0.05 (two-tailed), **p < 0.01 (two-tailed); correlations are statistically significant. Correlation coefficients are rounded to two decimal places; only the lower triangular matrix is displayed.

### 3.1 Significant parallel mediators: political participation exhibits a stable positive mediating effect, while political communication shows dimensional heterogeneity

According to Hayes’s (2018) guidelines for testing mediation effects, after including mediating variables, if the 95% confidence interval for the direct effect (path *c*′) includes 0, it is classified as full mediation; if the confidence interval does not include 0, it is classified as partial mediation. If the lower limit of the confidence interval is exactly 0, it is considered marginally significant. After including five parallel mediating variables, the 95% confidence intervals for the direct effects of all eight political identity dimensions did not include 0, indicating that the entire identity framework exhibits stable partial mediating effects. Only the emotional identity dimension exhibited marginal partial mediation, with the lower limit of its direct effect confidence interval exactly equal to 0. As shown in Table 4, political participation acted as a significant positive mediator, while the effects of political communication diverged markedly across identity clusters, with the two behavioral mediators exhibiting entirely distinct transmission patterns. Specific findings are as follows:

**Table 4:** Summary of multiple parallel mediating effects of eight dimensions of political identity (*N* = 4,094).

Identity dimension	Total effect (*β*)	Direct effect (*β* [95% CI])	Rights cognition (rig_m) (*β* [95% CI])	Political participation (par_m) (*β* [95% CI])	Political communication (com_m) (*β* [95% CI])	Benefit cognition (ben_m) (*β* [95% CI])	Political satisfaction (sat_m) (*β* [95% CI])	Total mediation effect (*β* [95% CI])
Identity (ide_m)	0.101	0.043 [0.025, 0.061]***	0.038 [0.026, 0.051]***	0.014 [−0.000, 0.027]*	0.001 [−0.012, 0.015]	−0.021 [−0.026, −0.017]***	0.026 [0.013, 0.039]***	0.058 [0.039, 0.077]***
Emotional (emo_m)	0.090	0.015 [0.000, 0.030]*	0.030 [0.020, 0.041]***	0.030 [0.016, 0.045]***	−0.001 [−0.013, 0.012]	−0.017 [−0.020, −0.013]***	0.032 [0.019, 0.046]***	0.075 [0.057, 0.095]***
Institutional (ins_m)	0.349	0.114 [0.089, 0.140]***	0.046 [0.030, 0.064]***	0.139 [0.118, 0.161]***	0.047 [0.026, 0.068]***	−0.032 [−0.039, −0.026]***	0.034 [0.015, 0.053]***	0.235 [0.209, 0.261]***
Systemic (sys_m)	0.261	0.041 [0.018, 0.064]***	0.053 [0.037, 0.070]***	0.087 [0.067, 0.110]***	0.047 [0.026, 0.068]***	−0.025 [−0.030, −0.019]***	0.057 [0.038, 0.078]***	0.220 [0.193, 0.247]***
Party ID (par_id)	0.188	0.052 [0.027, 0.077]***	0.057 [0.039, 0.075]***	0.054 [0.033, 0.077]***	0.010 [−0.012, 0.032]	−0.032 [−0.039, −0.026]***	0.047 [0.025, 0.068]***	0.136 [0.107, 0.165]***
Cultural ID (cul_id)	0.297	0.105 [0.076, 0.134]***	0.072 [0.054, 0.092]***	0.086 [0.063, 0.109]***	0.030 [0.006, 0.056]**	−0.036 [−0.044, −0.029]***	0.040 [0.017, 0.063]***	0.192 [0.160, 0.225]***
Policy ID (pol_id)	0.495	0.130 [0.096, 0.164]***	0.096 [0.074, 0.119]***	0.138 [0.109, 0.167]***	0.090 [0.059, 0.121]***	−0.038 [−0.048, −0.030]***	0.079 [0.053, 0.106]***	0.365 [0.331, 0.401]***
Developmental ID (dev_id)	0.289	0.069 [0.041, 0.096]***	0.082 [0.063, 0.101]***	0.064 [0.041, 0.088]***	0.049 [0.026, 0.072]***	−0.040 [−0.048, −0.033]***	0.065 [0.042, 0.091]***	0.220 [0.190, 0.251]***

*p < 0.05, **p < 0.01, ***p < 0.001 (two-tailed). CI, confidence interval.

First, political participation exhibits a universal positive mediating effect. As shown in Table 4, political participation has significant positive indirect effects across all eight dimensions of political identity—identity, emotional, institutional, systemic, party, cultural, policy, and development—with standardized mediation coefficients ranging from 0.014 to 0.138; Among these, policy identity (*β* = 0.138, *p* < 0.001) and institutional identity (*β* = 0.139, *p* < 0.001) exhibited the strongest mediating effects. This indicates that participatory behaviors such as online policy advocacy and digital deliberation can enhance young people’s sense of political efficacy and strengthen their perception of procedural legitimacy, thereby boosting political identity across all dimensions.

Second, the mediating effect of political communication exhibits significant stratified heterogeneity. As shown by the significance of each path in Table 4, the mediating effect of political communication is not significant (*p* > 0.05) in the emotional-traditional cluster (identity, emotional, and cultural identity) and the political party identity dimension. This suggests that one-way official digital propaganda is insufficient to meet young people’s demands for equal dialog and struggles to foster deep emotional connections. Within the institutional performance cluster (institutional, systemic, policy, and development identity), political communication exhibits a significant positive mediating effect (*β* = 0.030–0.090, *p* < 0.01). It can be anticipated that two-way digital channels focused on institutional and development information can effectively enhance identity at the governance level; conversely, superficial exposure to official propaganda alone has limited effectiveness. This highlights that the public’s perception of actual governance outcomes is the key foundation underpinning party identity.

### 3.2 Hierarchical sensitivity spectrum of the three dimensions of political identity

Based on the comparison of mean mediation values across clusters in Table 5, there are systematic hierarchical differences in the sensitivity of the three clusters to digital mediation pathways.

**Table 5:** Comparison of mediation effect intensity across clusters (*N* = 4,094).

Cluster classification	Number of dimensions	Mean direct effect (*β*)	Mean total mediation effect (*β*)	Mean rights cognition (*β*)	Mean political participation (*β*)	Mean political communication (*β*)	Mean benefit cognition (*β*)	Mean political satisfaction (*β*)
Affective-Traditional Cluster (Identity, Emotion, Culture)	3	0.054	0.108	0.047	0.043	0.010	−0.025	0.033
Institutional-Performance Cluster (Institutional, System, Policy, Development)	4	0.089	0.260	0.069	0.107	0.058	−0.034	0.059
Party Identification	1	0.052	0.136	0.057	0.054	0.010	−0.032	0.047

β represents the standardized regression coefficient, reflecting the number of standard deviation units the dependent variable (each dimension of recognition) changes when the independent variable (digital intelligence technology awareness) increases by 1 standard deviation. A larger numerical value indicates higher impact intensity.

#### 3.2.1 Emotional-traditional cluster (identity, emotional, and cultural identity)

As shown by the cluster means in Table 5, the total mean mediation effect for this cluster is only 0.108, the lowest among the three groups. Perceptions of rights and satisfaction with public services are two stable, positive mediators, while the mediating strength of political participation is relatively weak (mean 0.043), and political communication has no significant mediating effect. The underlying logic suggests that young people’s sense of belonging to their country and ethnic group is rooted in the actual experience of rights protection and public services; mere digital participation and online propaganda are insufficient to foster deep emotional identification.

#### 3.2.2 Institutional performance cluster (institutional, systemic, policy, and developmental identity)

The mean total mediating effect for this cluster is 0.260, far higher than that of the other two categories, making it the core vehicle through which digital technology exerts its role in political socialization. Both behavioral mediators—political participation and political communication—exhibit strong positive effects, while the negative inhibitory effect of perceived interests is equally prominent. Institutional and governance-related identities are highly dependent on the participatory and informational experiences provided by digital channels.

#### 3.2.3 Party identity (independent single dimension)

The mean total mediating effect of party identity is 0.136, falling between the two major clusters; satisfaction with public services is the strongest positive mediator (*β* = 0.047, *p* < 0.001), political participation has a moderate positive effect, and political communication has no significant mediating effect, reflecting the unique logic of legitimacy transformation in party identity, which centers on perceptions of the ruling party’s performance.

### 3.3 Stable positive drive and significant inhibition mediation: the consistent role of perceptions of rights and interests

In addition to the two behavioral mediators—political participation and political communication—perceptions of rights and interests exhibit a consistent negative effect across all dimensions of identity, forming a unified drive-masking mechanism. See Table 4 for complete data.

Perception of rights mediates in a consistently positive manner across all dimensions: mediation coefficients for the eight identity dimensions range from 0.030 to 0.096, all of which are significantly positive. The more fully young people perceive their digital political rights and channels for defending their rights, the more their sense of political belonging and evaluations of the political system improve in tandem; this serves as a stable, foundational driving force in shaping youth identity.

Perception of interests acts as a significantly inhibitory mediator across all dimensions: mediation coefficients range from −0.017 to −0.040, consistently exerting a masking effect. When young people perceive a disconnect between their personal demands and individual interests and national development, political identity at all levels is weakened; satisfaction with public services can offset this negative effect to a certain extent.

### 3.4 Comprehensive conclusions on the overall model’s total effects

Based on a comprehensive analysis of the overall effects across all dimensions in Table 4 and the cluster-stratified means in Table 5, perceptions of digital and intelligent technologies do not exert a uniform direct effect on the eight dimensions of young people’s political identity. All effects rely on five parallel cognitive/behavioral mediators to form a composite transmission chain. The complete causal pathway is: perceptions of digital and intelligent technologies → perceptions of rights/political participation/satisfaction with public services (positive), political communication (positive across dimensions), and perceptions of interests (negative inhibitory effect) → stratified and differentiated political identity.

Empirical findings do not support the research perspective that digital technology is a “neutral tool”: The effects of digital technology on the political socialization of young people do not depend on the technology itself, but rather on the political participation channels, two-way communication mechanisms, and information on the dissemination of rights embedded in digital environments, as well as differences in individual young people’s perceptions of their interests. At the same time, political identity exhibits stable, stratified heterogeneity: institutional performance-based identity is more easily fostered positively through digital channels, while emotional and party-based identities rely more heavily on deep-seated, real-world perceptions such as rights protection and public services; therefore, purely online information campaigns yield limited results.

## 4 Discussion

### 4.1 Analysis of the underlying mechanisms of multiple mediating pathways

The results of the multiple parallel mediation analysis show that political participation plays a significant positive mediating role in the process by which perceptions of digital technology influence the eight dimensions of political identity. The standardized coefficients of the mediating effects range from 0.014 to 0.139, making political participation the significant transmission channel across all dimensions of identity; In stark contrast, political communication exhibits a significant positive mediating effect only within the institutional performance cluster (institutional, systemic, policy, and developmental identity). Its mediating effect is not significant in the three emotionally rooted identity dimensions—identity, emotional, and party—and thus fails to form an effective transmission pathway. The differing mechanisms underlying the roles of these two types of mediating variables can be explained by drawing on theories such as the theory of digital participation empowerment, the theory of psychological resistance, and the theory of social identity.

The theory of digital participation empowerment and related research indicate that young people’s use of digital channels to proactively gather policy information, participate in online public discussions, and voice social concerns can continuously enhance their sense of political efficacy, thereby strengthening their recognition of the nation, its institutions, and its developmental achievements (Goncalves et al., 2026; Lai and Beh, 2024; Zhang et al., 2024). Data from 4,094 samples in this study show that the correlation coefficient between digital technology literacy and political participation is as high as 0.61. The higher the digital literacy of college students, the stronger their willingness to actively participate in online political practices; sustained participation continuously reinforces their macro-level identification with the system and development achievements. The theories of psychological resistance and social identity offer plausible explanations for the heterogeneous outcomes of political communication. A key mechanism of the theory of psychological resistance is that when individuals perceive their behavioral freedom or right to choose as being threatened by external forces, they enter a state of motivational arousal aimed at restoring their lost freedom (Carpenter et al., 2026). According to Tajfel and Turner’s social identity theory, individuals integrate group membership into their self-concept through social categorization and social comparison, thereby internalizing norms (Huddy, 2001). However, when this internalization process is perceived as a threat to individual freedom or autonomy, psychological resistance mechanisms are activated, leading individuals to adopt defensive reactions to restore their perceived freedom, thereby blocking or reversing the internalization of identity (Ma et al., 2019). One-way government propaganda and push-style policy outreach targeting young people constitute passive information intake; when the frequency of information pushes is too high and the content is highly homogeneous, young people are prone to developing psychological resistance. This defense mechanism, rooted in psychological resistance, effectively blocks the process of internalizing social identity. For identity, emotional, and party affiliations rooted in personal emotions and a sense of cultural belonging, one-way indoctrination is perceived as a deprivation of autonomy, triggering negative evaluations of the “indoctrinated group” and thereby eroding deep emotional connections. Consequently, passive digital communication struggles to resonate with underlying emotional logic and fails to serve as a mediating factor. Conversely, for institutional identities centered on rational performance evaluations, standardized government information can intuitively demonstrate governance outcomes, and one-way communication can still effectively enhance levels of identification. This precisely explains the data pattern whereby political communication is effective only when institutional performance clusters are in effect.

Based on existing research on digital politics among young people in China, most of the current literature either examines a single mediating pathway of digital participation in isolation (Yang, 2020; Zheng, 2017) or broadly combines the two categories of digital behaviors—information communication and political participation (Liu et al., 2023; Wang and Luo, 2018). These studies fail to distinguish between the differentiated transmission effects of active participation and passive, one-way communication (Zhou and Liu, 2018), making it difficult to reveal the stratified effects of digital behaviors on different types of political identity (Tao and Zhang, 2022; Zhang and Guo, 2020). This study decomposes political participation and political communication into two independent and parallel mediating pathways, clearly identifying the boundaries of their respective roles, thereby addressing the shortcomings of existing research characterized by coarse variable categorization and ambiguous path mechanisms. Another counterintuitive finding of theoretical significance in this study is that perceived interests exhibit a stable and significant negative mediating effect across all eight dimensions of political identity, with standardized mediation coefficients ranging from −0.040 to −0.017, making it the only channel that consistently inhibits political identity. Descriptive statistics reveal that interest perception is the only variable among all variables with a mean below 3 points (*M* = 2.63) and a standard deviation as high as 1.505, indicating an extremely high degree of evaluation divergence within the sample. From the perspective of the digital information echo chamber theory, prolonged exposure to homogeneous information and viewpoints tends to create a closed, monolithic information environment. This phenomenon not only limits users’ opportunities to access diverse perspectives but may also exacerbate social polarization, weaken critical thinking, and pose a challenge to rational dialog in the public sphere (Piao et al., 2023; O’Reilly et al., 2024). Analyzing the internal logic of political identity through the lens of the digital information bubble theory reveals that digital platform algorithms tend to push content aligned with individuals’ personal demands and narrow self-interest. Young people who remain confined to a narrow, micro-level perspective of personal interests over the long term are prone to exaggerating disparities in local interests during the development process, leading to a one-sided perception of interests. Students with higher digital literacy have access to more diverse information regarding various interests and are better able to identify developmental imbalances, which in turn reduces their sense of personal benefit; conversely, a low level of interest awareness weakens various forms of political identity, ultimately forming a negative feedback loop: “increased digital literacy → decreased interest awareness → weakened political identity.” This negative pathway counteracts the three positive pathways—perception of rights, political participation, and political satisfaction—constituting a classic masking effect. It also fully demonstrates that the process by which digital technology shapes young people’s political identity is not a one-way benefit; rather, it involves an internal interplay between positive and negative forces. This contrasts with previous approaches that primarily relied on single or chain-based mediators, or that aggregated identity dimensions into a single composite score (Zheng, 2017; Zhou and Liu, 2018; Tao and Zhang, 2022), and it also demonstrates that failing to disaggregate dimensions leads to an underestimation of path heterogeneity (see Table 5).

### 4.2 Explanation of the characteristics of stratified heterogeneity in the transmission of political identity dimensions

Based on the three major clusters—emotional tradition, institutional performance, and party identity—established in this study, a comparison of the mediating effects across clusters clearly reveals significant stratified differences: the average total mediating effect for the institutional performance cluster is 0.260, approximately 2.4 times the mean of 0.108 for the emotional tradition cluster, while the total mediating effect for the party identification cluster (0.136) falls between the two. Stable differentiation exists across all three clusters in terms of direct effects and the strength of individual mediating pathways, reflecting fundamental differences in the ways digital technology shapes different types of political identity.

The underlying logical differences between these two types of core cluster identities profoundly validate the explanatory power of social Identity theory and cognitive mobilization theory as the two pillars for understanding the psychological mechanisms of contemporary political participation. Specifically, the emotional tradition cluster follows the logic of social Identity theory. This cluster encompasses identity, emotion, and cultural identity, rooted in young people’s long-standing sense of belonging to their national culture and their innate emotional attachment to their homeland. Just as this theory emphasizes the foundational role of group belonging and in-group preference in motivating action (Kelly, 1993), this type of identity is endogenous and emotionally driven. Data show that this cluster exhibits a higher proportion of direct effects, indicating that digital technology relies more on subtly influencing innate emotions through direct perception and struggles to profoundly alter deep-seated psychological structures and identity-related emotions via mediating variables. In contrast, the institutional performance cluster embodies the core mechanisms of cognitive mobilization theory. This cluster encompasses institutional, systemic, and developmental identities, relying on young people’s rational and objective evaluations of the effectiveness of national governance. This corresponds to the process by which individuals, through enhanced information-processing capabilities, reduce their reliance on traditional authority and instead engage rationally in politics through non-traditional channels (such as digital platforms) (Poletti, 2015), representing an exogenous, performance-driven form of identity. The data show that the mean values of all positive mediating pathways in the institutional performance cluster are consistently higher than those in the traditional emotional cluster; specifically, the average mediating effect for political participation is 0.107, far exceeding the 0.043 observed in the emotional cluster. This suggests that digital cognition and behavior exert a stronger influence on shaping identity based on rational evaluation (the cognitive mobilization dimension).

This study introduces a new binary cluster stratification perspective and, by combining five parallel mediating pathways, deconstructs the transmission differences. This essentially represents an extension of the “jointly driven by emotion and reason” mechanism within the theory of local political identity structures. At the same time, the data from this study effectively address the limitations of mainstream Western theories on algorithmic polarization. Western theories argue that algorithms amplify divisions and fragment identity (Bakshy et al., 2015; Ford, 2025), but this perspective does not align well with China’s hybrid digital ecosystem. This study finds that perceptions of digital technology generally predict all dimensions of political identity positively, and no fragmentation of identity has emerged. Examining the local factors, domestic digital platforms serve dual functions of policy promotion and public communication, while algorithmic recommendations balance macro-level narratives with individual demands. Furthermore, unobstructed channels for online civic participation allow young people to voice their concerns and engage in governance through digital channels, thereby offsetting the anxiety over localized interests generated by algorithms. This unique transmission model fully reflects the local heterogeneity of the mechanisms through which political identity is shaped by digital technologies.

### 4.3 Dialog with previous research and theoretical innovations of this study

#### 4.3.1 Engaging with Western literature on digital technology and political identity to provide counterevidence from local experiences

Western academia has developed two mainstream theoretical perspectives regarding digital technology and the formation of public political attitudes: the theory of technological neutrality holds that digital tools themselves do not possess value biases; they are morally and politically neutral—neither good nor evil—and their value depends entirely on how humans use them (Miller, 2020). Therefore, they have no fixed positive or negative effect on public identity; The theory of digital polarization, on the other hand, emphasizes that algorithms and social media platforms exacerbate social divisions and continuously erode public trust in institutions and national identity (König and Wenzelburger, 2021; Trenz, 2023). Both theoretical frameworks are constructed based on the social context of Western multiparty competition and media commercialization, and their explanatory power is clearly limited by geographical constraints.

Based on the results of multiple mediation analyses conducted on a sample of 4,094 Chinese college students, this study provides direct evidence to the contrary: at the aggregate level, the overall effect of digital technology cognition on all eight dimensions of political identity is significantly positive; there is no evidence of a general erosion of political identity or polarization and social fragmentation, and the study does not support the universality of Western theories of digital polarization; At the same time, digital technology is not entirely neutral; its impact on political identity is jointly determined by five distinct mediating pathways, involving both stable positive transmission channels and negative inhibitory channels. The direction of these effects depends on the complex interplay of mediating psychological and behavioral variables, making it impossible to simply label digital technology as neutral or solely negative.

#### 4.3.2 Benchmarking against domestic research on youth political socialization to address the limitations of dimensional homogenization

Although research on youth political identity in China has evolved from establishing theoretical frameworks to exploring mediating mechanisms (Shi et al., 2014; Zhang and Guo, 2020), the widespread limitations of dimensional homogenization and singular pathways have long obscured the stratified shaping effects of digital technologies.

This study deconstructs political identity into eight distinct dimensions and classifies them into three clusters based on their intrinsic logic. By testing parallel multiple mediation models dimension by dimension and cluster by cluster, it clearly identifies the differentiated boundaries of influence for variables such as political communication, political participation, and perception of interests. The study proposes the core argument that “the impact of digital technology on political identity involves a dimensional stratification mechanism,” thereby overcoming the shortcomings of previous research—namely, dimensional homogeneity and overly general conclusions— refining the stratified research perspective on the political socialization of youth and enriching the empirical research paradigm on the political identity of Chinese youth.

#### 4.3.3 Extension of the theoretical model: constructing a complete causal chain of “cognition of digital and intelligent technologies—multiple mediators—layered and multidimensional political identity”

Most existing studies in this field employ simple chain mediation or single-mediator models, which can only explain a single transmission pathway, making it difficult to capture the complex psychological processes—including cognition, behavior, and perception of interests—among young people in a digital environment. Furthermore, few studies combine a stratified identity structure with multiple parallel mediators, failing to fully illustrate the complex interactive relationships among variables.

This study integrates theories of digital participation empowerment, psychological resistance, the information bubble, and social Identity theory to construct a comprehensive theoretical model comprising 1 independent variable, 5 parallel mediators, and 8 hierarchical dependent variables, thereby establishing a complete causal framework spanning the entire chain: “Perceptions of Digital and Intelligent Technologies → Perceptions of Rights/Political Participation/Political Communication/Perceptions of Interests/Satisfaction with Public Services → Hierarchical Multidimensional Political Identity.” The model simultaneously accounts for both commonalities in unified transmission and dimensional heterogeneity: on the one hand, it identifies universal patterns—namely, the stable positive mediation of rights perception and political satisfaction, and the stable negative mediation of interest perception; on the other hand, it distinguishes transmission differences across emotional, institutional, and party clusters, balancing unified mechanisms with stratified characteristics. By combining multiple parallel mediation analyses with the stratified structure of political identity, this model expands the analytical framework in the fields of digital politics and the political socialization of youth, providing a reference framework and theoretical approach for future empirical research on similar multidimensional constructs and multi-path transmission mechanisms.

### 4.4 Implications for research and practice

Based on the findings regarding mediating mechanisms and hierarchical heterogeneity in this study, this paper proposes targeted optimization strategies for digital ideological and political education in higher education institutions and online government publicity efforts. First, prioritize expanding channels for young people’s proactive digital political participation; campus public opinion surveys and digital government feedback platforms can fully leverage their significant positive mediating role in political participation, thereby continuously strengthening young people’s rational recognition toward the system and development. Second, optimize one-way digital government publicity formats. Reduce homogeneous, push-style messaging regarding young people’s emotional and party identity dimensions; instead, employ soft communication strategies that incorporate cultural narratives and emotional stories to avoid psychological resistance effects; Third, balance the supply of information on digital platforms. While showcasing developmental achievements, reasonably address young people’s specific interest-based demands to alleviate the one-sided perception of interests caused by information silos and weaken the negative inhibitory effect of interest-based cognition; Fourth, implement tiered and differentiated digital ideological and political guidance: for institutional performance-based recognition, emphasize rational dissemination of policy data and governance outcomes; for emotional and traditional recognition, focus on immersive digital content featuring national culture and patriotic sentiments; and conduct precise value-oriented guidance based on the characteristics of tiered communication.

## 5 Limitations and future research

This study still has certain limitations: the sample consists solely of college students, making it difficult to directly generalize the findings to young adults in the general population; furthermore, the use of cross-sectional survey data prevents the identification of dynamic, time-series causal relationships among variables. Future research could expand the sample to include young adults in the general population and those engaged in new economic sectors, and conduct longitudinal tracking studies. It could also incorporate additional moderator variables to test the moderating effects of factors such as digital usage duration, media trust, and ideological and political education in schools within the model. Additionally, by combining qualitative interviews, researchers could delve deeper into the underlying psychological motivations behind the negative mediating effect of perceived benefits and the heterogeneous role of political communication, thereby further refining the stratified transmission theory of how digital technology influences young people’s political identity.

## Data Availability

The original contributions presented in the study are included in the article/Supplementary material, further inquiries can be directed to the corresponding author.
