# Supplementary material for: The heterogeneous effects of perceptions of digital and intelligent technologies on multidimensional political identity among college students: a study of multiple parallel mediations from a stratified cluster perspective
Source: Front Psychol. 2026 Oct 22;17:1794810. doi: 10.3389/fpsyg.2026.1794810 (PMC13735778; doi:10.3389/fpsyg.2026.1794810)
Supplement: Supplementary file 1 [file Table_1.pdf]

Supplementary Material

The heterogeneous effects of perceptions of digital and intelligent technologies on multidimensional political identity among college students: a study of multiple parallel mediations from a stratified cluster perspective

Qian Hou

School of Marxism, Shandong University of Technology, Zibo, China

Frontiers in Psychology

2026

Supplementary Material

This file provides the supplementary tables cited in Section 2.2 (Measures) of the manuscript: **Supplementary Table S1** (the complete measurement instrument, comprising the 28 multi-item entries and the single-item anchor measures) and **Supplementary Table S2** (internal consistency reliability for all multi-item scales, N = 4094, including Cronbach's α, the corrected item–total correlations, and the item-treatment decisions).

Supplementary Table S1 Measurement Items of Research Variables

Item ID	Item Content	Options
**Political Identity (Dependent Variable)**		
**Identity Identification (Multi-item, 3 items)**		
Q1-1	As a Chinese in the new era, I feel very proud.	1 = Strongly Agree ~ 5 = Strongly Disagree
Q1-2	When mistaken for Korean/Japanese, I will solemnly state that I am Chinese.	Same as above
Q1-3	My identity as a Chinese citizen is crucial to me.	Same as above
**Emotional Identification (Multi-item, 3 items)**		
Q2-1	Feelings toward the motherland.	1 = Highly Affectionate ~ 5 = Completely Unaffectionate
Q2-2	Feelings toward the Chinese nation.	Same as above
Q2-3	Feelings toward the Communist Party of China.	Same as above
**Institutional Identification (Multi-item, 3 items)**		
Q4-1	The political system of reform and opening-up has greatly promoted national development.	1 = Strongly Agree ~ 5 = Strongly Disagree
Q4-2	The development of New China demonstrates the superiority of the socialist system with Chinese characteristics.	Same as above
Q4-3	Your sense of participation and gain under China's political system.	1 = A Great Deal ~ 5 = Very Little
**Systemic Identification (Multi-item, 3 items)**		
Q3-1	Level of understanding of the political system of socialism with Chinese characteristics.	1 = Very Well Understood ~ 5 = Completely Ununderstood
Q3-2	The degree of alignment between the political system of socialism with Chinese characteristics and personal values.	1 = Highly Aligned ~ 5 = Completely Misaligned
Q3-3	The advantages of the political system of socialism with Chinese characteristics compared to Western political systems.	1 = Clearly Superior ~ 5 = Clearly Inferior
**Cultural Identification (Single-item, Anchor)**		
Q6-1	Excellent traditional Chinese culture has a significant impact on individuals.	1 = Strongly Agree ~ 5 = Strongly Disagree
**Party Identification (Single-item, Anchor)**		
Q5-1	Adhering to the leadership of the Party is crucial for China's development.	Same as above
**Policy Identification (Single-item, Anchor)**		
Q7-1	The policies of the Party and the government in the new era are suitable for China's actual development.	Same as above
**Developmental Identification (Single-item, Anchor)**		
Q8-1	Reform and opening-up is the right choice for China's modernization process.	Same as above

Supplementary Table S1 (continued) Measurement Items of Research Variables

Item ID	Item Content	Options
**Independent Variable**		
**Digital Intelligence Technology Cognition (Multi-item, 7 items)**		
Q9-1	Level of understanding of digital intelligence technologies.	1 = Very Well Understood ~ 5 = Completely Ununderstood
Q9-2	Digital intelligence technologies are widely applied across various industries.	1 = Strongly Agree ~ 5 = Strongly Disagree
Q9-3	Digital intelligence technologies are developing rapidly towards deeper integration, enhanced computing power, and generalized intelligence.	1 = Strongly Agree ~ 5 = Strongly Disagree
Q9-4	Digital intelligence technologies have limitations and risks such as technical ethics and data security.	1 = Strongly Agree ~ 5 = Strongly Disagree
Q9-5	Digital intelligence technologies have had a significant impact on life, work, study, etc.	1 = Strongly Agree ~ 5 = Strongly Disagree
Q9-6	Digital intelligence technologies bring social risks such as information cocoons, group polarization, and algorithmic black boxes/discrimination.	1 = Strongly Agree ~ 5 = Strongly Disagree
Q9-7	Current college students urgently need to improve their capabilities and literacy in acquiring, processing, and utilizing digital intelligence technology-related information.	1 = Strongly Agree ~ 5 = Strongly Disagree

Supplementary Table S1 (continued) Measurement Items of Research Variables

Item ID	Item Content	Options
**Mediating Variables**		
**Rights Cognition (Multi-item, 3 items)**		
Q10-1	Chinese citizens have a very strong awareness of their rights.	1 = Strongly Agree ~ 5 = Strongly Disagree
Q10-2	National development requires the expansion of citizens' rights.	Same as above
Q10-3	The state needs to improve in safeguarding citizens' rights.	Same as above
**Benefit Cognition (Single-item, Anchor, Reverse-coded)**		
Q10-4	Among all interests, personal interest is the most important.	Same as above
**Political Participation (Multi-item, 3 items)**		
Q10-5	Concern about national political news and policy trends.	1 = Very Concerned ~ 5 = Completely Unconcerned
Q10-6	China's development requires the political participation of college students.	1 = Strongly Agree ~ 5 = Strongly Disagree
Q10-7	The Party and government provide various effective channels for college students' political participation.	1 = Strongly Agree ~ 5 = Strongly Disagree
**Political Communication (Multi-item, 3 items)**		
Q10-8	Communication between the government and the public can effectively resolve problems and contradictions.	1 = Strongly Agree ~ 5 = Strongly Disagree
Q10-9	Only with full access to information can effective political communication be achieved.	Same as above
Q10-10	The Party and government provide various communication channels for college students.	Same as above
**Political Satisfaction (Single-item, Anchor)**		
Q10-11	Very satisfied with the public policies and services provided by the government.	1 = Strongly Agree ~ 5 = Strongly Disagree

Supplementary Table S2 Summary of Internal Consistency Reliability for All Multi-item Scales (N = 4094)

Latent Variable / Dimension	Number of Items	Cronbach's α	Range of Corrected Item-Total Correlation (CITC)	Item Treatment Decision
Identity Identification	3	0.715	0.494 ~ 0.585	All items retained; all CITC > 0.3; deleting any item would reduce overall α.
Emotional Identification	3	0.810	0.592 ~ 0.756	All items retained; excellent reliability with high item aggregation.
Systemic Identification	3	0.651	0.429 ~ 0.577	Acceptable range; all CITC met standards; all items retained.
Institutional Identification	3	0.666	0.426 ~ 0.603	Acceptable range; no inefficient items; all items retained.
Rights Cognition	3	0.770	0.562 ~ 0.647	All items retained; deleting any item would reduce overall reliability.
Political Participation	3	0.755	0.557 ~ 0.608	All items retained; deleting any item would reduce overall reliability.
Political Communication	3	0.761	0.510 ~ 0.650	All items retained; deleting a single item only marginally increased α; full retention prioritized to maintain construct coverage.
Digital Tech. Cognition	7	0.889	0.571 ~ 0.776	All items retained; deleting any item reduced overall reliability; excellent scale aggregation.
Global Aggregate (All 28 Items)	28	0.925	0.262 ~ 0.706	All 28 multi-item entries aggregated; single-item anchor variables excluded from calculation; overall reliability is excellent.

Notes.

1. This table summarizes the reliability of multi-item scales only. Single-item measures (Party Identification, Cultural Identity, Policy Support, Development Recognition, Benefit Cognition, and Public Service Satisfaction) do not calculate Cronbach's α and are excluded from this table.
2. Reliability criteria: Cronbach's α > 0.70 indicates good reliability; 0.60 ~ 0.70 is the acceptable threshold for applied research. A CITC > 0.3 indicates that an item adequately aggregates with its dimension.
3. The last row presents the global reliability index after aggregating all multi-item entries. The statistical scope differs from the dimension-specific reliabilities listed above, as it excludes single-item constructs such as Party Identification and Cultural Identity.
